# RNAi as a Tool to Study Virulence in the Pathogenic Yeast *Candida glabrata*

**DOI:** 10.3389/fmicb.2019.01679

**Published:** 2019-07-24

**Authors:** Olena P. Ishchuk, Khadija Mohamed Ahmad, Katarina Koruza, Klara Bojanovič, Marcel Sprenger, Lydia Kasper, Sascha Brunke, Bernhard Hube, Torbjörn Säll, Thomas Hellmark, Birgitta Gullstrand, Christian Brion, Kelle Freel, Joseph Schacherer, Birgitte Regenberg, Wolfgang Knecht, Jure Piškur

**Affiliations:** ^1^Department of Biology, Lund University, Lund, Sweden; ^2^Department of Biology and Biological Engineering, Systems and Synthetic Biology, Chalmers University of Technology, Gothenburg, Sweden; ^3^Department of Microbial Pathogenicity Mechanisms, Hans Knöll Institute, Jena, Germany; ^4^Institute of Microbiology, Friedrich Schiller University, Jena, Germany; ^5^Department of Nephrology, Lund University, Lund, Sweden; ^6^Department of Molecular Genetics, Genomics and Microbiology, Strasbourg University, Strasbourg, France; ^7^Department of Biology, Faculty of Science, University of Copenhagen, Copenhagen, Denmark; ^8^Lund Protein Production Platform, Lund University, Lund, Sweden

**Keywords:** *Candida glabrata*, pathogenic yeast, RNA interference, RNAi, gene library, antifungal drugs, virulence factors, macrophages

## Abstract

The yeast *Candida glabrata* is a major opportunistic pathogen causing mucosal and systemic infections in humans. Systemic infections caused by this yeast have high mortality rates and are difficult to treat due to this yeast’s intrinsic and frequently adapting antifungal resistance. To understand and treat *C. glabrata* infections, it is essential to investigate the molecular basis of *C. glabrata* virulence and resistance. We established an RNA interference (RNAi) system in *C. glabrata* by expressing the Dicer and Argonaute genes from *Saccharomyces castellii* (a budding yeast with natural RNAi). Our experiments with reporter genes and putative virulence genes showed that the introduction of RNAi resulted in 30 and 70% gene-knockdown for the construct-types antisense and hairpin, respectively. The resulting *C. glabrata* RNAi strain was used for the screening of a gene library for new virulence-related genes. Phenotypic profiling with a high-resolution quantification of growth identified genes involved in the maintenance of cell integrity, antifungal drugs, and ROS resistance. The genes identified by this approach are promising targets for the treatment of *C. glabrata* infections.

## Introduction

The yeast *Candida glabrata* is an opportunistic human pathogen that causes relatively benign mucosal or fatal systemic infections. The incidence of infections caused by this *Candida* species has significantly increased particularly in immune-deficient patients, in addition to those that undergo chemotherapy, treated with broad-spectrum antibiotics for prolonged time, or have undergone a higher number of invasive surgeries (Pfaller and Diekema, 2007). In healthy humans, *Candida* species colonize the oral cavity, vagina, and gut (Mårdh et al., 2002; Moyes and Naglik, 2011). In many studies, *C. glabrata* and *C. albicans* are considered the most common causes of candidiasis (Fidel et al., 1999; Brunke and Hube, 2013). While *C. glabrata* is a distant relative of *C. albicans*, it is a close relative of bakers’ yeast *Saccharomyces cerevisiae* (Dujon et al., 2004). In evolutionary time scales, *C. glabrata* only “recently” became pathogenic (Brunke and Hube, 2013; Ahmad et al., 2014). The mechanisms behind *C. glabrata* pathogenicity are thus far not well understood, but appear to differ greatly from *C. albicans*. Immune evasion strategies, possibly via intracellular survival and replication in macrophages contribute to the virulence of *C. glabrata* (Seider et al., 2011, 2014; Brunke and Hube, 2013; Kasper et al., 2014). *C. glabrata* also readily rearranges its genome (Ahmad et al., 2014) at a frequency one order of magnitude higher than in *C. albicans* (Gabaldón and Fairhead, 2018). Genome rearrangements could be evolutionary advantageous for this yeast, allowing it to adapt rapidly to its host environment and to antifungal treatments.

Research focused on *C. glabrata* pathogenicity can benefit greatly from the development of molecular tools. In *S. cerevisiae*, the strategy of gene deletion has been used for decades as an approach to study gene function. However, Giaever et al. (2002) found that approximately 1000 mutants (representing ∼17% of all *S. cerevisiae* genes) failed to grow as a result of a specific gene deletion in haploids, proving that these genes are essential for this yeast. The genome of *C. glabrata* has 5283 predicted coding sequences, and extensive research has been devoted to the creation of a whole genome deletion library for this haploid yeast (Schwarzmüller et al., 2014). Large-scale gene deletions for *C. glabrata* proved less feasible than for *S. cerevisiae* due to gene essentiality or technical reasons (lower homologous recombination rates). However it must be assumed that *C. glabrata* contains a similar number of essential genes as *S. cerevisiae.*

Other techniques, such as down-regulating gene expression can provide alternative ways to study genes’ functions. The RNA interference (RNAi), which relies on manipulating the levels of a gene’s transcript, has become a widespread tool to analyze the function of genes in organisms ranging from protozoa to human. Unlike gene deletions, RNAi can be applied to any gene, even those that are essential. It has, however, not found many applications in baker’s yeast, which naturally lack important components of the RNAi machinery (Drinnenberg et al., 2009). The RNAi approach relies on the activity of two proteins, Dicer (ribonuclease III) and Argonaute (the carrier of small interfering RNA [siRNAs]). The process is initiated by the cleavage of double stranded RNA (complementary to the target transcript) by Dicer into siRNAs, which are then loaded on Argonaute and, by base-pair interaction, target the gene’s transcript. This ultimately interferes with gene expression by down-regulation. RNAi is found widely in nature (plants, animals, and fungi), and provides evolutionary advantages by protecting these organisms from viruses, taking part in gene silencing, heterochromatin organization, and chromosome segregation (Tijsterman et al., 2002; Martienssen et al., 2005; Moazed, 2009).

Remarkably, although RNAi is well preserved among some fungi (Drinnenberg et al., 2009), it has been lost in several budding yeasts such as *S. cerevisiae* and *C. glabrata*. The aim of this study was to develop a RNAi system for *C. glabrata* and to use it as a tool to identify and functionally analyze genes known to be putatively required for virulence.

## Results

### Introduction of RNAi Into *C. glabrata*

**FIGURE 1:**
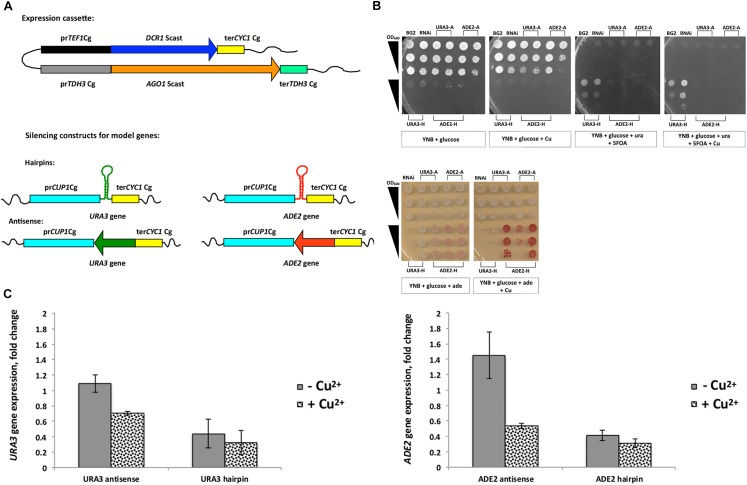
Introduction of RNA interference (RNAi) into *C. glabrata.* **(A)** Schematic view of the plasmid carrying the expression cassettes for the *DCR1* and *AGO1* genes of *S. castellii* (P1062), which was integrated into the genome; the *DCR1* cassette consisted of the promoter *TEF1* and the terminator *CYC1* of *C. glabrata;* the *AGO1* cassette consisted of the promoter *TDH3* and the terminator *TDH3* of *C. glabrata;* the silencing constructs (antisense and hairpins) targeting two model genes of the *C. glabrata* genome, *URA3* and *ADE2*, were expressed under control of the inducible promoter *CUP1* with the terminator *CYC1*, both of *C. glabrata.* **(B)** Growth phenotypes of *C. glabrata* transformant carrying RNAi constructs for *URA3* and *ADE2* genes (hairpin [H] and antisense [A]). Serial dilutions of cells (OD_600_ 1; 0.1; 0.01) were plated on different media with and without copper (YNB; YNB with adenine; YNB with 5-FOA and uracil); Strains are as follows: 1 – BG2; 2 – Y1662 carrying P1028 (RNAi negative control); 3 – *URA3* antisense-8; 4 – *URA3* antisense-12; 5 – *ADE2* antisense-5; 6 – *ADE2* antisense-10; 7 – *URA3* hairpin-4; 8 – *URA3* hairpin-7; 9 – *ADE2* hairpin-3; 10 – *ADE2* hairpin-4; 11 – *ADE2* hairpin-10. **(C)** Expression analysis of *ADE2* and *URA3* genes in RNAi transformants performed by qRT-PCR. Gene expression was induced by addition of copper to the medium. Strains: control (Y1662 carrying P1028); *ADE2* antisense; *ADE2* hairpin; *URA3* antisense; *URA3* hairpin. The error bars represent standard deviation of two replicates. Corresponding primer pairs were used with cDNA: *ACT1* gene (OP33 and OP34), *ADE2* (OP31 and OP32), *URA3* (OP27 and OP28).

For the reconstitution of an RNAi mechanism in *C. glabrata*, two heterologous *Saccharomyces castellii* genes, *DCR1* and *AGO1* (coding for Dicer and Argonaute, correspondingly) were introduced into the *C. glabrata* genome. For this purpose we first evaluated the activity of several constitutive and inducible promoters by their ability to induce expression of a functional enzyme, deoxyribonucleoside kinase of *Drosophila melanogaster* (Supplementary Figure S1, Methods). The *dNK* gene encoding this enzyme was successfully expressed in *C. glabrata* and different enzyme activity was observed when expressed from different promoters (Supplementary Figure S1). For the expression of *DCR1* and *AGO1* genes in the *C. glabrata*, we used the strong constitutive promoters *TEF1* and *TDH3* of *C. glabrata* (Figure 1A and Supplementary Figure S1). The plasmid carrying the *DCR1* and *AGO1* constructs (P1062, Supplementary Table S2) was linearized by *Sph*I and integrated into the genome of three standard laboratory *C. glabrata* strains, BG2, CBS138 and BG14 (Table 1).

**Table 1:** Strains used in this study.

Laboratory designation	Original name	Origin	Description	Source
Y1092	CBS 138 (ATCC 2001)		Wild type	Reference strain
Y1630	BG2	Clinical isolate	Wild type	(Fidel et al., 1996) Kindly provided by Cormack B.
Y1638	BG14	BG2	*ura3*	(Cormack and Falkow, 1999) Kindly provided by Cormack B.
Y1636	Y1092 *lys2*	CBS 138 (Y1092)	*lys2*	This study
Y1637	BG2 *lys2*	BG2	*lys2*	This study
Y1662	BG2 *lys2 DCR1 AGO1*	BG2	RNAi master strain, *lys2* carrying P1062 (stable transformant)	This study
Y1699	Y1092 *lys2 DCR1 AGO1*	CBS 138	RNAi master strain, *lys2* carrying P1062 (stable transformant)	This study
Y2121	BG14 ura3 *DCR1 AGO1*	BG14	RNAi master strain, *ura3* carrying P1062 (stable transformant)	This study
Y1663	Y1637 *DCR1 AGO1 URA3* antisense-8	Y1662	Y1662 carrying P1029	This study
Y1664	Y1637 *DCR1 AGO1 URA3* antisense-12	Y1662	Y1662 carrying P1029	This study
Y1665	Y1637 *DCR1 AGO1 URA3* hairpin-4	Y1662	Y1662 carrying P1030	This study
Y1667	Y1637 *DCR1 AGO1 URA3* hairpin-7	Y1662	Y1662 carrying P1030	This study
Y1660	Y1637 *DCR1 AGO1 ADE2* antisense-5	Y1662	Y1662 carrying P1065	This study
Y1661	Y1637 *DCR1 AGO1 ADE2* antisense-10	Y1662	Y1662 carrying P1065	This study
Y1671	Y1637 *DCR1 AGO1 ADE2* hairpin-3	Y1662	Y1662 carrying P1066	This study
Y1672	Y1637 *DCR1 AGO1 ADE2* hairpin-4	Y1662	Y1662 carrying P1066	This study
Y1673	Y1637 *DCR1 AGO1 ADE2* hairpin-10	Y1662	Y1662 carrying P1066	This study
Y1843	Y1699 *DCR1 AGO1 PUP1* antisense	Y1699	Y1699 carrying P1125	This study
Y2172	Y1699 *DCR1 AGO1 PUP1* hairpin	Y1699	Y1699 carrying P1151	This study
Y1847	Y1637 *DCR1 AGO1*	Y1662	Y1662 carrying empty P1061	This study
Y1848	Y1636 *DCR1 AGO1*	Y1699	Y1699 carrying empty P1061	This study


To first prove that the RNAi pathway can function in *C. glabrata*, we generated silencing constructs targeting the two genes *URA3* (encoding orotidine 5′-phosphate decarboxylase) and *ADE2* (encoding phosphoribosylaminoimidazole carboxylase) in the *C. glabrata* genome. They are part of the uracil (*URA3* gene) and adenine (*ADE2* gene) biosynthesis pathway and are popular auxotrophic markers in yeast genetics. Lack or loss of function of these genes products result in following phenotypes: requirement of uracil/adenine for growth, accumulation of *p*-ribosylaminoimidazolecarboxylate (red pigment) in the absence of *ADE2* transcript, and resistance to 5-FOA without *URA3*. We used two different silencing constructs for these genes (antisense and hairpin), which we introduced into the *lys2* auxotrophic variant of BG2 carrying RNAi genes (Y1662, Table 1) by transforming it with integrative plasmids. The design of silencing constructs was similar to that used by Drinnenberg et al. (2009), were the antisense constructs consisted of 339 bp of antisense DNA strand of the gene and hairpins had 339 bp of both antisense and sense DNA strand separated by 79 bp “loop” (Supplementary Data). *Sal*I linearized the plasmids with antisense constructs, and *Sma*I linearized hairpin plasmids before the transformation. For the expression of silencing constructs we selected the strong inducible *C. glabrata* promoter *CUP1* (Figure 1A and Supplementary Figure S1).

Two to three selected transformants of the RNAi strain carrying different silencing constructs were grown on different selective media (Figure 1B). We observed a decreased growth among transformants carrying antisense constructs (*URA3, ADE2*) under conditions of *CUP1* promoter induction (medium supplemented with copper) on minimal medium lacking uracil or adenine. For hairpin constructs (*URA3, ADE2*), a growth inhibition by several orders of magnitudes was observed with and without *CUP1* promoter induction. Apparently, the *CUP1* promoter was leaky (Supplementary Figure S1) and the hairpin constructs had a strong effect on gene expression. In agreement with this, silencing of the *URA3* gene with the hairpins allowed growth on 5-FOA (Figure 1B), a compound, which is toxic to cells with an active *URA3* gene. Further, *ADE2* silencing with hairpins resulted in accumulation of the red pigment *P*-ribosylamino imidazole on adenine-limited media (10 mg/L), which indicates a block in adenine synthesis (Figure 1B). These results show a strong silencing of model target genes by both *URA3* and *ADE2* hairpin constructs.

To confirm our phenotypic observations, the expression of *URA3* and *ADE2* genes was studied by qRT-PCR in transformants carrying either antisense or hairpin silencing constructs (Figure 1C). Compared to the control strain, both *ADE2* antisense and *URA3* antisense constructs caused 1.4-times and 2-times down-regulation, respectively, of their target genes specifically in the medium with copper (Figure 1C). In contrast, gene expression in transformants with hairpin constructs was repressed further than that even in the medium without copper, with only slight additional increase in the presence of Cu ions (Figure 1C).

The experiment showed that the reconstituted RNAi pathway in *C. glabrata* is functional and can be applied for gene silencing in this yeast.

### Knock-Down of a Known Virulence Gene

In addition to model genes, we designed silencing constructs for *PUP1/*CAGL0M12947g gene of *C. glabrata* to be tested in our RNAi system in *C. glabrata* CBS 138 strain. The deletion of this gene, encoding a mitochondria localized protein, decreases the virulence of azole resistant strain DSY565 of *C. glabrata* in an immuno-compromised mouse model (Vermitsky et al., 2006; Ferrari et al., 2011). Both DSY565 and CBS 138 strains are fluconazole resistant. As estimated in our lab, the CBS138 strain has fluconazole MIC of 129 mg/L, which is higher than that of DSY565 (fluconazole MIC of 64 mg/L, Vale-Silva et al., 2017). To achieve a stable integration for silencing constructs in the genome, we targeted the 18S rDNA locus of *C. glabrata* CBS 138 strain with RNAi. For a constitutive expression level, the promoter *PGK1* of *C. glabrata* was selected (Supplementary Figure S1).

We constructed two recombinant plasmids carrying antisense or hairpin constructs for the *PUP1* (Supplementary Table S2). These vectors (P1125 and P1151) were linearized with *SacII* (this restriction site is present in the 18S rDNA sequence) and transformed into CBS 138-based *C. glabrata* master strain Y1699 carrying both *DCR1* and *AGO1* genes (Table 1). The plasmid linearization in 18S rDNA region generated homologous regions for ends-in homologous recombination with 18S rDNA loci. To check the stability of the plasmids’ integration into the genome, the recombinant colonies were grown for several generations on non-selective YPD medium. The strains were analyzed by PCR to confirm the presence of antisense or hairpin “bullets” and integration of both *DCR1* and *AGO1*. Then 90% of all strains carrying the inserts were stable for 60 generations.

**FIGURE 2:**
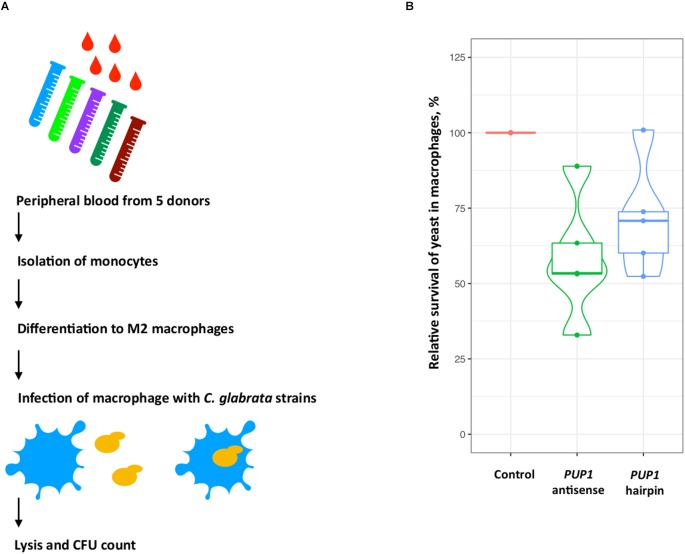
In *C. glabrata*, silencing-constructs of the putative virulence-associated gene *PUP1* (CAGL0M12947g) inhibit yeast-survival in the macrophage model. **(A)** M2 macrophages differentiated from peripheral-blood monocytes of five donors were infected with the three *C. glabrata* strains. Yeast survival was assessed by CFU count. **(B)** The relative survival-rates of *C. glabrata* strains carrying antisense and hairpin constructs of the *PUP1* gene were determined and normalized to the control strain carrying an empty vector (Y1848, set as 100% survival and used to normalize data). The plot is based on the data of five replicates for each construct.

Using this approach, we constructed two strains, Y1843 and Y2172, which carry antisense and hairpin constructs for the *C. glabrata PUP1* gene, respectively. As survival in macrophages may be one important virulence determinant of *C. glabrata* (Brunke and Hube, 2013), we used a macrophage-confrontation assay for the analysis of fitness of our mutant. In this macrophage confrontation assay, the strains carrying the *PUP1* antisense and hairpin constructs showed decreased intracellular survival of 42% (for antisense construct) and 29% (for hairpin construct) of their mean values compared to the empty-vector control strain Y1848 (Figure 2). While the silencing constructs reduced survival comparing to the control (ANOVA, *p* = 0.02 and *p* = 0.1), the difference between the antisense and hairpin transformants survival was not significantly different (ANOVA, *p* = 0.42). Compared to the control strain, the *PUP1* gene expression was 10-fold lower in the *PUP1* antisense strain, and 1000-fold lower in *PUP1* hairpin strain (Supplementary Figure S2). The ratio of gene size to antisense region was ∼2. Our results of the down regulation of *PUP1* gene by silencing constructs prove that our RNAi system can be used to study gene functions in an infection model and show that *PUP1* is required for *C. glabrata* survival in macrophages.

### Construction of RNAi Gene Library to Detect New Virulence-Associated Genes of *C. glabrata*

**FIGURE 3:**
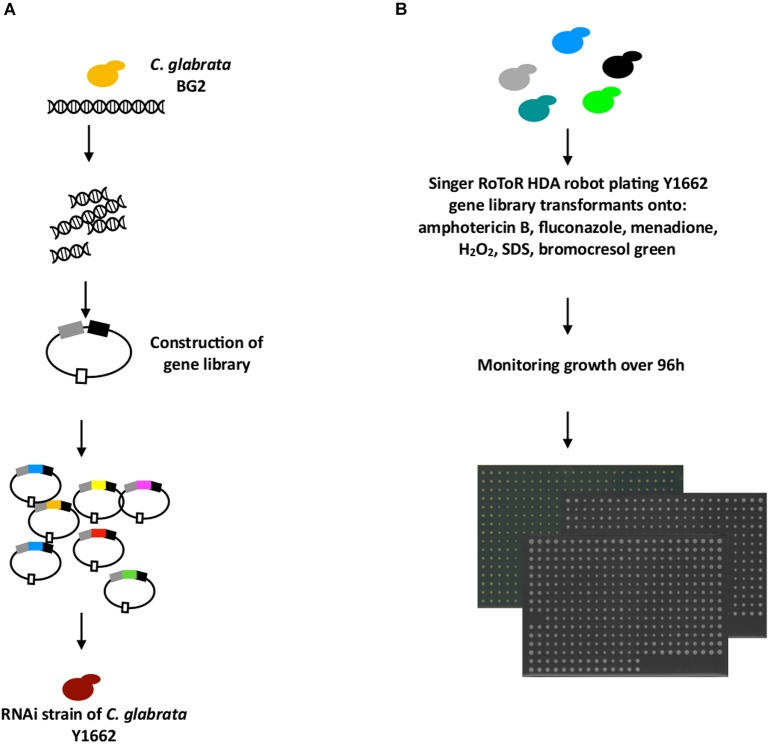
Cloning of putative virulence genes from a gene library. **(A)** Genomic DNA for gene library construction was isolated from the *C. glabrata* BG2 strain. The DNA was partially digested with *Sau*3AI and cloned into the *C. glabrata* multi-copy expression vector P1226 under control of the *TEF1* promoter and with *CYC1* terminator. This library was transformed into the RNAi-capable *C. glabrata* strain Y1662. **(B)** 1080 transformants of Y1662 containing gene library plasmids were plated from liquid YNB onto different media of interest (amphotericin B, fluconazole, menadione, hydrogen peroxide, SDS, bromocresol green) using a robotic system in 384-colony format plates. The growth of colonies was monitored by the digital imaging over 96 h.

To use our RNAi system as a basis for the identification of infection-relevant *C. glabrata* genes, we constructed a library of *C. glabrata* genome fragments on a plasmid vector (P1226) carrying an ARS-like sequence, which results in about 10 copies per cell (Hanic-Joyce and Joyce, 1998) (Figure 3A). The expression cassette for genomic fragments contained the strong constitutive promoter *TEF1* and the terminator *CYC1* of *C. glabrata*. The resulting gene-library plasmids were isolated from 400,000 bacterial transformants, and 40% of these carried inserts ranging from 1 to 5 kb in size as estimated by *Sau*3AI enzyme digestion of the plasmid DNA. The constructed gene library therefore represented at least a 10× genome coverage of *C. glabrata* as estimated by the number of clones and their plasmid insert sizes. This library was used to transform the BG2-based *C. glabrata* strain with the reconstituted RNAi pathway, Y1662 (Figure 3). In this case, both sense and antisense DNA fragments (depending on the orientation of the ligated genomic fragments) could determine the transformant’s phenotype. We expected that the sense fragments lead to gene up-regulation, while the antisense fragments lead to gene down-regulation (silencing).

### RNAi Gene Library Phenotypic Screening Identifies Putative Virulence-Associated Genes

A total of 1080 *C. glabrata* strains with the gene library were selected randomly from ∼5000 colonies obtained after transformation, and subjected to phenotypic profiling in search of novel virulence-related genes involved in ROS tolerance, and antifungal drug resistance (Figure 3), attributes that help *C. glabrata* survive in the host. In addition, we selected medium with SDS for the large-scale cloning of genes responsible for *C. glabrata* cell integrity, and pH indicator for monitoring the colonies surface pH (Figure 3). Later can be explored further as targets for the development of new antifungals to destroy the cell or studied for its pH alteration properties in macrophages.

**FIGURE 4:**
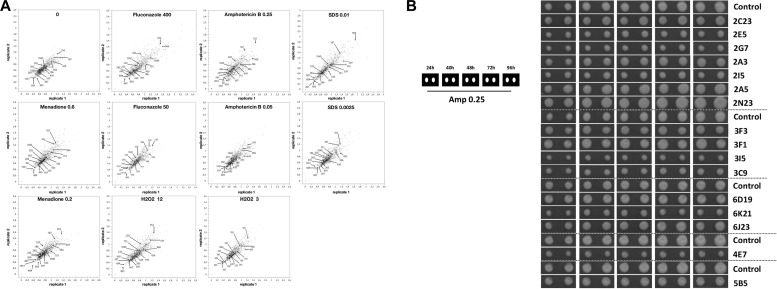
The gene library transformants had different growth phenotypes. **(A)** Relative colony size of two replicates of 1080 gene library transformants of the RNAi-capable strain under different conditions estimated at 48 h of incubation. Colony size of transformants with the library was normalized to the control (Y1662 with empty vector, which was set to 1.0) on each plate. Conditions: 0 (no treatment), antifungals (fluconazole [50 and 400 μg/ml], amphotericin B [0.05 and 0.25 μg/ml]), ROS-generating compounds (hydrogen peroxide [3 and 12 mM], menadione [0.02 and 0.06 mM]), and surfactant (SDS, 0.0025 and 0.01%). **(B)** Growth for 96 h of transformants (two replicates each) selected for sequencing on the medium with AmB (0.25 μg/ml).

In the first round of screening, 1080 transformants were analyzed for their growth capacity in two replicates at high resolution using a robotic platform. The plating and growth screening were carried out on solid media containing fluconazole, amphotericin B, menadione, hydrogen peroxide, SDS, or the pH indicator bromocresol green. As shown in Figure 4, the *C. glabrata* transformant colonies varied in their growth phenotypes with a high correlation between two replicates. In comparison to the control medium (YNB with 2% glucose), the addition of selected compounds in most conditions resulted in more divergent transformant phenotypes (colony sizes) relative to the control transformant with an empty vector (used for the normalization and has value 1). Transformants with colony size different to the control strain under the conditions studied are of interest for further study, as the genes, which can contribute to this superior or diminished growth, are potential antifungal targets. Several transformants were resistant or sensitive to different stress conditions (Figure 4).

The second round of screening was performed for the sensitivity to the potent new antifungal peptides 6, 9, and 11 developed by Larsen et al. (2015). For this, we used selected transformants with stress-related phenotypes (identified in the first round, 139 transformants) and 54 previously untested randomly picked up transformants. Transformants were tested in replicate and 11 transformants proved to be sensitive or resistant to one or all of the antifungal peptides tested (Table 2).

**Table 2:** Sequencing analysis of gene library plasmids.

Plasmid name from robot screening	Plasmid name from manual screening	Gene present on the plasmid insert	Gene description (www.candidagenome.org)	Insert orientation	Phenotype
2C23, #220	81E18	CAGL0L00157g	Uncharacterized, homologous to other adhesion-like GPI-anchored proteins of *C. glabrata* (*EPA11, EPA19*, etc.).	Antisense	Fluconazole (-) Amphotericin B (-) Menadione (-) pH (modified) SDS (-)
2E5, #235	81C18	CAGL0E00231g	Uncharacterized, putative adhesin-like protein; contains tandem repeats and a predicted GPI-anchor; belongs to adhesin cluster III.	Antisense	Fluconazole (-) Amphotericin B (-) Menadione (-) SDS (-) H_2_O_2_ (-)
2G7, #260	81E14	CAGL0I11011g	Uncharacterized, putative adhesin; belongs to adhesin cluster V.	Antisense	Fluconazole (-) Amphotericin B (-) Menadione (-) SDS (-) H_2_O_2_ (-)
2A3, #186	81E12	CAGL0B01683g	*TMN2*, uncharacterized, ortholog(s) have role in cellular copper ion homeostasis, invasive growth in response to glucose limitation, pseudohyphal growth, vacuolar transport and fungal-type vacuole membrane localization.	Sense	Fluconazole (-) Menadione (-) SDS (-) pH (modified) Peptide 9 (+) H_2_O_2_ (-) SDS (-)
2I5, #283		CAGL0G05335g	*TPS2*, uncharacterized, ortholog(s) have trehalose-phosphatase activity.	Antisense	Fluconazole (-) Amphotericin B (-) Menadione (-) SDS (-) H_2_O_2_ (-)
5N2, #893	81C6	CAGL0J02464g	Uncharacterized, Ortholog(s) have SUMO-specific isopeptidase activity, role in chromosome condensation, mitotic spindle assembly checkpoint, plasmid maintenance, protein desumoylation, and nucleus localization.	Antisense	Fluconazole (-) Amphotericin B (-) Menadione (-) SDS (-) Peptide 6 (-) Peptide 9 (-) Peptide 11 (-) H_2_O_2_ (-)
3F3, #430	81C4	CAGL0K11968g	Uncharacterized, ortholog(s) have 3-hydroxyisobutyryl-CoA hydrolase activity and mitochondrial ribosome localization.	Antisense	Fluconazole (-) Amphotericin B (-) Menadione (-) SDS (-) pH (modified) H_2_O_2_ (-)
2A5, #187	81E4	CAGL0H05049g	Uncharacterized, ortholog(s) have leucine-tRNA ligase activity, mRNA binding activity, role in Group I intron splicing, leucyl-tRNA aminoacylation, mitochondrial translation and mitochondrion localization.	Sense	Fluconazole (+) Amphotericin B (+) Menadione (+) SDS (+) Peptide 9 (+) H_2_O_2_ (+)
2N23, #352	81C22	CAGL0D05588g	Uncharacterized, ortholog(s) have role in maturation of SSU-rRNA from tricistronic rRNA transcript (SSU-rRNA, 5.8S rRNA, LSU-rRNA) and nucleolus, small-subunit processome localization.	Sense	Fluconazole (+) Amphotericin B (+) H_2_O_2_ (+) Menadione (+) SDS (+)
	81N8	CAGL0A01430g	Uncharacterized, putative tryptophan synthase; protein abundance increased in *ace2* mutant cells.	Antisense	Amphotericin B (-)
6D19, #966	81A16	CAGL0E00539g	Uncharacterized, ortholog(s) have role in attachment of spindle microtubules to kinetochore involved in homologous chromosome segregation, chromatin silencing at rDNA, protein localization to nucleolar rDNA repeats, rDNA condensation.	Antisense	Fluconazole (+) Amphotericin B (+) SDS (+) Peptide 6 (+) Peptide 9 (+) Peptide 11 (+)
6K21, #1051	81A14	CAGL0J01661g	Uncharacterized, has domain(s) with predicted transmembrane transporter activity and role in transmembrane transport.	Sense	Fluconazole (-) Amphotericin B (-) Menadione (-) SDS (-) H_2_O_2_ (-)
3F1, #429		CAGL0M04741g	Uncharacterized, ortholog(s) have protein disulfide isomerase activity, protein disulfide oxidoreductase activity, protein-disulfide reductase (glutathione) activity and role in protein folding.	Sense	Amphotericin B (+) SDS (+)
4E7, #604		CAGL0K01991g	Uncharacterized, has domain(s) with predicted tRNA (cytosine-5-)-methyltransferase activity.	Sense	Fluconazole (-) Amphotericin B (-) Menadione (-) SDS (-) H_2_O_2_ (-)
5B5, #751	81G5	CAGL0J03652g	Uncharacterized, has domain(s) with predicted ATP binding, aminoacyl-tRNA editing activity, leucine-tRNA ligase activity, role in leucyl-tRNA aminoacylation and cytoplasm localization.	Sense	Fluconazole (-) Amphotericin B (-) Peptide 9 (-)
6J23, #1040		CAGL0A03630g	Uncharacterized, ortholog(s) have RNA polymerase III general transcription initiation factor activity, chromatin insulator sequence binding activity and role in transcription initiation from RNA polymerase III promoter.	Sense	Fluconazole (-) Amphotericin B (-) Menadione (-) H_2_O_2_ (-)
3I5, #467	81I15	CAGL0I07843g	*ADH1*, Putative alcohol dehydrogenase isoenzyme III; increased protein abundance in azole resistant strain.	Sense	Fluconazole (-) Amphotericin B (-) Menadione (-) SDS (-) Peptide 6 (-) Peptide 9 (-) Peptide 11 (-) H_2_O_2_ (-)
3C9, #397	81I21	CAGL0A00781g	Uncharacterized, has domain(s) with predicted zinc ion binding activity.	Sense	Fluconazole (-) Amphotericin B (-) Menadione (-) SDS (-) Peptide 9 (-) H_2_O_2_ (-)
	82H20	CAGL0B05049g	Uncharacterized, ortholog(s) have ubiquitin-protein transferase activity and role in double-strand break repair via non-homologous end joining, double-strand break repair via synthesis-dependent strand annealing.	Sense	Amphotericin B (-) Peptide 6 (-) Peptide 9 (-) Peptide 11 (-)
	82N22	CAGL0H00891g	Uncharacterized, ortholog(s) have actin filament binding, calmodulin binding activity.	Antisense	Amphotericin B (-) Peptide 6 (-) Peptide 9 (-) Peptide 11 (-)
	81E20	CAGL0G05335g	*TPS2*, uncharacterized, ortholog(s) have trehalose-phosphatase activity.	Antisense	Amphotericin B (-)
	82M12	CAGL0L13392g	Protein of unknown function.	Sense	Amphotericin B (-) Peptide 6 (+) Peptide 9 (+) Peptide 11 (+)
	83K4	CAGL0D06446g	*STT4*, uncharacterized, ortholog(s) have 1-phosphatidylinositol 4-kinase activity.	Sense	Fluconazole (+)
	83K11	CAGL0H07623g	Uncharacterized, ortholog(s) have role in meiotic gene conversion, reciprocal meiotic recombination.	Antisense	Amphotericin B (-) Peptide 6 (-)


*“#”, location on the graph, Figure 4. “+”, resistant. “-”, sensitive.*

In total, we randomly selected 82 gene library transformants for sequencing, all of which showed increased or decreased stress sensitivity, and/or changed resistance to antifungal drugs tested, for sequencing. The inserts of the isolated plasmids were sequenced from the promoter *TEF1* and the terminator *CYC1* and 24 inserts sequences, which were obtained in full, contained one gene (or portion of a gene) in either the sense or anti-sense orientation (Table 2). The *C. glabrata* CAGL0G05335g gene, (*TPS2*) encoding a subunit of trehalose-6-phosphate synthase/phosphatase complex and known to be associated with virulence in *C. albicans* (Van Dijck et al., 2002), was found on two plasmids. Most of the genes isolated were so far uncharacterized (Table 2). Potential gene functions predicted form orthologs of identified genes in other species cover numerous cellular processes including RNA processing and transcription regulation, adhesins, trehalose biosynthesis, ethanol production, protein folding, chromosome maintenance and recombination, and metabolite transport. We also found two clones with antisense fragments to genes, whose orthologs in *S. cerevisiae* are essential: CAGL0H00891g (*S. cerevisiae IQG1* encodes protein required cytokinesis (Epp and Chant, 1997), and CAGL0A01430g [*S. cerevisiae TRP5* gene encodes tryptophan synthase and whose mutations results in tryptophan auxotrophy (Braus, 1991)].

**FIGURE 5:**
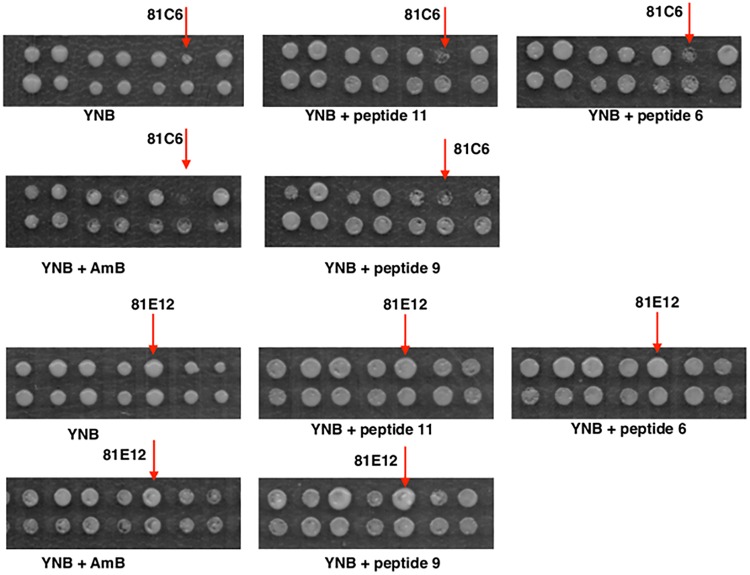
Growth of gene library transformants of RNAi strain on antifungal peptides (#6, #9, #11) and amphotericin B (AmB). The transformants, which displayed sensitivity or resistance to the compounds tested were selected for sequencing (*arrowed*).

Seven transformants proved to be sensitive to one or to all antifungal peptides tested (Table 2). For example, transformant 81C6 (5N2) was sensitive to peptide 6, 9, and 11 (Table 2 and Figure 5), and was found to carry the plasmid with antisense fragment to the *C. glabrata* CAGL0J02464g gene, which encodes a putative isopeptidase, that is possibly able to directly cleave or modify the tested peptides. Four transformants proved to be resistant to one or to all antifungal peptides tested (Table 2). Transformant 81E12 (2A3) was resistant to peptide 9 (Table 2 and Figure 5), and was found to carry the plasmid with sense fragment of the *C. glabrata* CAGL0B01683g/*TMN2* gene’s open reading frame, which ortholog in *S. cerevisiae* is a membrane protein, which take part in endosome-vacuolar trafficking. The degradation of other antimicrobial peptides by targeting to yeast vacuole was reported by other studies (Lis et al., 2009; Muñoz et al., 2013).

**FIGURE 6:**
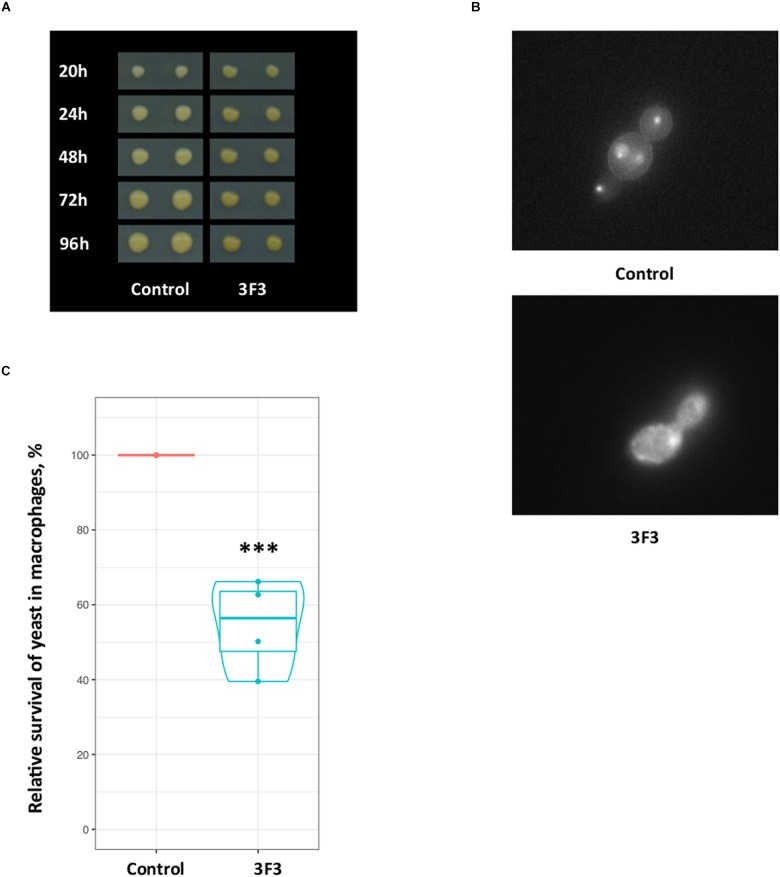
*Candida glabrata* CAGL0K11968g contributes to vacuolar morphology and survival in macrophages. **(A)** On the medium with pH indicator bromocresol green the 3F3 strains had darker yellow color than control strain, indicating more acidic pH. **(B)** Fluorescent microscopy images of *C. glabrata* strains after staining showing abnormal vacuolar morphology of the 3F3 strain. **(C)** The transformant 3F3, which carries an antisense CAGL0K11968g gene, displayed 50% loss of viability in our macrophage model. The experiment was performed in four replicates with cells originating from blood of two donors. The plot is based on data of four replicates. The control strain data (CFU) was set as 100% and used to normalize data. According to Poisson Regression analysis of raw data, the 3F3 strains CFU response was significantly different (^∗∗∗^*p*-value ≤ 0.0005).

In addition sensitivity to antifungal drugs, the transformant 3F3 displayed more acidic colony surface on medium with pH indicator bromocresol green, as the colonies of this strain had darker yellow color than the control strain (Figure 6A), indicating acid pH (≤3) of 3F3 colonies. The observed properties of 3F3 on bromocresol green could affect its survival after phagocytosis. Sequencing of the insert of the gene library plasmid isolated from 3F3 showed that it carried an antisense insert of the uncharacterized gene of CAGL0K11968g of *C. glabrata*. In *S. cerevisiae*, the gene-ortholog has a 3-hydroxyisobutyryl-CoA hydrolase activity and a role in the stress-activated MAPK cascade with its mutation affecting the fluid-phase endocytosis^1^. Indeed, vacuolar staining revealed that as in deletion mutants of *S. cerevisiae*, the down-regulation of this gene in *C. glabrata* affects the vacuolar morphology (Figure 6B). We tested the 3F3 strain in our macrophage model, where it was 50% more susceptible to killing after macrophage phagocytosis (Figure 6C). The macrophages phagosome maturation is accompanied with high level of ROS, and one of *C. glabrata* strategies to proliferate inside the macrophages is lowering ROS (Seider et al., 2011). Our study of 3F3 strain showed that it had lowered proliferating properties inside the macrophages (lowered survival). The expression of the 3F3 plasmid (antisense) was also found to confer hydrogen peroxide sensitivity in RNAi strain but not in wild type (Supplementary Figure S3).

In summary, using high resolution of mitotic growth screening of gene library in RNAi strain we cloned several putative virulence-related genes.

### RNAi Gene Library Antisense Plasmids Inhibit the Expression of Their Target Genes

Further studies are needed to verify each isolated gene by the gene overexpression studies or gene deletions. Moreover, the antisense and truncated gene constructs may have off target effects on other gene expression as observed in mammalian cells (El-Brolosy and Stainier, 2017; Ma et al., 2019; Wilkinson, 2019), and studying the global gene expression of these strains could give more information on this in future studies. In this study, to rule-out the possibility of off-target gene inhibitions we have investigated the target gene expression of 10 antisense constructs in both wild type and RNAi strains.

**FIGURE 7:**
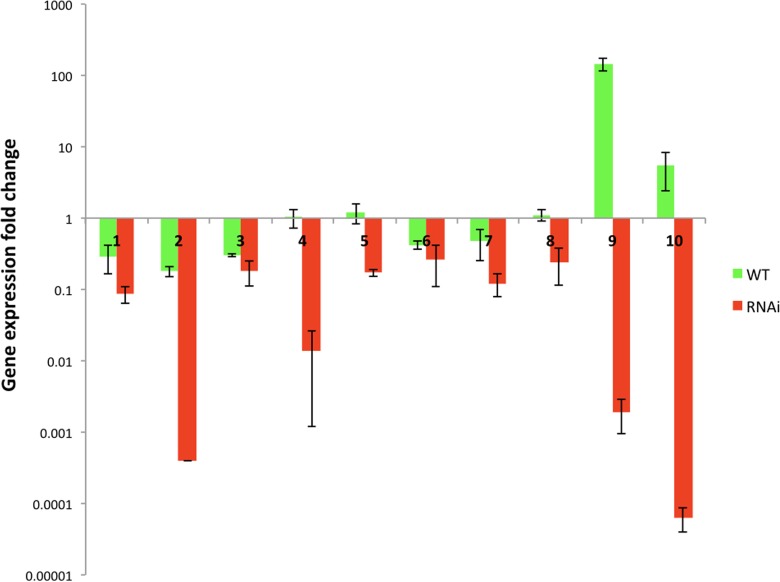
The target genes expression studies of the transformants of RNAi and wild type strain carrying antisense gene library plasmids. *Y*-axis – logarithmic scale. RNAi strain was Y1662, and the wild type was Y1637, which were newly re-transformed with plasmids. Antisense plasmids: 1 – 2C23 (CAGL0L00157g gene), 2 – 2E5 (CAGL0E00231g gene), 3 – 81E20 (CAGL0G05335g gene), 4 – 6D19 (CAGL0E00539g gene), 5 – 3F3 (CAGL0K11968g gene), 6 – 2I5 (CAGL0G05335g gene), 7 – 82N22 (CAGL0H00891g gene), 8 – 81N8 (CAGL0A01430g gene), 9 – 83K11 (CAGL0H07623g gene), and 10 – 2G7 (CAGL0I11011g gene). The untreated controls were used to calculate gene expression fold change: RNAi and wild type carrying empty vector (P1226). Primer pair ACT1-1 and ACT1-2 was used for *ACT1* gene.

For this purpose we took ten gene library antisense plasmids (2C23, 2E5, 81E20, 6D19, 3F3, 2I5, 82N22, 81N8, 83K11, and 2G7) and transformed them into the wild type and RNAi strains. Two transformants of each plasmid were selected for the analysis. The RNA was extracted from their cultures grown in YNB with 2% glucose and gene expression was studied by qRT-PCR (Figure 7 and Supplementary Figure S4). Plasmids 2C23, 2E5, 81E20, 2I5, and 82N22 had stronger gene expression inhibition in RNAi strain than in WT. Plasmids 6D19, 3F3, 81N8, 83K11, and 2G7 proved to inhibit the target genes in RNAi strain but not in the WT strain (Figure 7). This suggests that the gene inhibition is indeed mediated through RNAi. Phenotypes observed during the robotics screening were re-confirmed for most plasmids after the re-transformation (Supplementary Figure S3). While some of the phenotypes were unique to RNAi transformants (H_2_O_2_ sensitivity in 2I5, 3F3, 82N22, and 81E20), 2I5 conferred the amphotericin resistance to both RNAi and wild type strain (Supplementary Figure S3).

## Discussion

The yeast *C. glabrata* is an important opportunistic human pathogen, which has become of interest over recent decades, particularly due to its increasing occurrence and resistance to antifungal drugs.

Unlike for *S. cerevisiae* and *C. albicans*, there are no extensive genetics toolboxes available for *C. glabrata* and thus this yeast remains poorly studied. Gene deletions in this yeast for gene function elucidations are not easy to obtain. With a higher rate of non-homologous recombination in *C. glabrata* than that in *S. cerevisiae* (Cormack and Falkow, 1999; Corrigan et al., 2013), longer homology regions are required for more efficient gene knockouts in *C. glabrata*; haploid asexual nature of *C. glabrata* prohibits many techniques based on mating developed for *S. cerevisiae.* In this study, we developed a new tool for *C. glabrata* based on RNA interference (RNAi), which relies on gene silencing through double-stranded RNA intermediates and siRNA. This ancient mechanism of protection from foreign DNA and chromatin organization is lost in Hemiascomycetes (Axelson-Fisk and Sunnerhagen, 2006) with the exception of *Saccharomyces castellii* (Drinnenberg et al., 2009). We therefore cloned the *DCR1* and *AGO1* genes from *S. castellii* and overexpressed them in *C. glabrata* together with silencing constructs. Judging from our data on silencing of metabolic genes (endogenous *ADE2* and *URA3* genes), the introduced RNAi pathway is functional and can be applied to additional genes of interest in this yeast. We found that gene silencing was stronger with hairpin compared to antisense constructs in this study similarly to Drinnenberg et al. (2009). Although our data indicate that the *CUP1* promoter used for RNAi is leaky, the antisense constructs resulted in decreased gene expression and detectable phenotypes only upon its induction by copper. In contrast, the hairpin constructs displayed high inhibition of gene expression regardless of the *CUP1* promoter induction, which suggests that the designed hairpins are very effective in our system.

Our RNAi tool proved to be active when we tested it with an established virulence-associated gene CAGL0M12947g (*PUP1*) of *C. glabrata*, which was shown by deletion to be important for a survival in a mouse model (Ferrari et al., 2011). Here, we showed that down-regulation of this gene affects survival in human macrophages. Its ortholog (*RCI37*/*YIL077C*) is poorly studied in *S. cerevisiae*. Although it is known to localize in the mitochondria and interact with the respiratory chain (Morgenstern et al., 2017), the mechanistic connections of its *C. glabrata* ortholog to virulence remain unknown.

We were interested to apply the developed RNAi for the discovery of new virulence-associated genes of *C. glabrata.* We did this by using the RNAi strain with a gene library as the basis for screening of virulence-related phenotypes and the identification of responsible genes. To this end we created a library of random genome fragments in the expression vector. In contrast to hairpin constructs, this approach is feasible on the genomic level (the library represents 10-fold genome coverage), and we can assume that a significant portion of clones will carry the antisense regions of genes to induce RNAi. Since the inhibitory effect of the antisense constructs was less efficient than hairpins in our model and required a high expression level, the gene library was constructed using the strong constitutive *TEF1* promoter and a multi-copy vector.

Our primary interest was to discover genes, which affect *C. glabrata* resistance to antifungal drugs, and to stress conditions that *C. glabrata* may face during survival in macrophages. Therefore, we selected the following conditions for cell growth *in vitro*, which have overlap in their targets in yeast. In our screening we selected fluconazole and amphotericin B, which are both antifungal drugs currently in use against candidiasis, and which target the plasma membrane. Although the mechanism of action of many azole drugs and resistance to them are quite extensively studied (Vale-Silva and Sanglard, 2015), the molecular mechanism of amphotericin B action and resistance remains poorly understood. We also tested hydrogen peroxide and menadione since ROS are elevated upon amphotericin B exposure (Mesa-Arango et al., 2014), and are produced inside the macrophages phagosomes (Seider et al., 2014), and resistance against ROS is important for pathogen survival. SDS was tested to mimic damage to the plasma membrane. In addition, we used new antifungal drugs for the *C. glabrata* gene library screening including three peptidomimetics with an arginine-[β-(2,5,7-tri-tert-butylindol-3-yl)alanine]-arginine motif, which were recently developed (Larsen et al., 2015), and are effective against *S. cerevisiae* as well as *Zygosaccharomyces bailii*, known to spoil food. Interestingly, *C. glabrata* was previously shown to be resistant to these peptides (Larsen et al., 2015) and finding the resistance genes in pathogenic yeasts can help to develop potent antifungals further.

The frequent and large size differences of transformants colonies in our screening indicated that a broad range of genes was covered by the RNAi library strains. We selected representative resistant and sensitive transformants for sequencing of their plasmid inserts to determine the original genome loci. Using this approach, we found that several positive clones of our RNAi library corresponded to putative virulence-associated genes. This shows that our approach allows us to recover genes with relevance for *C. glabrata* pathobiology, which can be exploited further for the development of treatments for *C. glabrata* infections.

The gene down-regulation and expression of truncated genes might affect the expression of other genes in the genome, their genes network as described for mammalian cells (El-Brolosy and Stainier, 2017; Ma et al., 2019; Wilkinson, 2019). This effect, genetic compensation, is also common for gene knockouts in yeasts (He and Zhang, 2006; Teng et al., 2013). The expression of antisense constructs spanning the coding regions in *S. cerevisiae* from the plasmid vectors were reported to inhibit the *ATH1* and *CAR1* gene expression (Park et al., 2001; Jung and Park, 2005) suggesting the natural antisense interference without RNAi (Donaldson and Saville, 2012). The whole genome sequencing and global gene expression profiling can give more information on any off-target effects of isolated gene library clones in the future studies. When we analyzed ten antisense constructs, all 10 down-regulated the target genes, and 5 of 10 worked through the RNAi because they did not down-regulate the gene expression in the wild type strain without RNAi pathway.

Three of the genes identified in our screen were predicted glycosylphosphatidylinositol (GPI)-anchored proteins and adhesins (CAGL0L00157g, CAGL0E00231g, and CAGL0I11011g), and the expression of their antisense regions leads to sensitivity to fluconazole, amphotericin B, menadione, hydrogen peroxide, and SDS. GPI-anchored proteins are abundant membrane and cell wall proteins with multiple roles, and their biosynthesis is reportedly linked ergosterol biosynthesis and azole drug response, Ras signaling (Yadav et al., 2014). The GPI-anchored proteins are therefore important antifungal targets, and are in the focus of drug discovery studies (Mann et al., 2015). It is important to note that the antisense region of the 2C23 isolate of our study included a part of the GPI-anchored protein transmembrane domain (CAGL0L00157g), which is a fragment of 17 other GPI-genes sequences in the *C. glabrata* genome. This suggests that the construct affected several GPI proteins, likely resulting in altered plasma membrane structure.

The 6K21 strain, which was sensitive to antifungals and ROS, carried the overexpression plasmid for a sense fragment of transporter gene CAGL0J01661g, whose ortholog in *S. cerevisiae* (YPR011C) encodes a mitochondrial transporter for adenosine 5′-phosphosulfate (APS) and 3′-phospho-adenosine 5′-phosphosulfate (PAPS), the deletion of whish causes the decreased glutathione and methionine levels and temperature sensitive phenotype (Todisco et al., 2014), and glutathione is known ROS scavenger (Jamieson, 1998).

Our results point at the *TPS2* gene, encoding trehalose-6 phosphate phosphatase of *C. glabrata*, as a potential drug target with virulence-associated roles in this yeast. The *TPS2* antisense construct expression resulted in sensitivity to fluconazole, amphotericin B, and other stressors. The deletion of *TPS2* in both *S. cerevisiae* and *C. albicans* causes the accumulation of increased amounts of trehalose-6 phosphate upon stress, which is toxic to the cell, and results in thermo-sensitive phenotype (De Virgilio et al., 1993; Van Dijck et al., 2002). In *C. albicans*, it was also found to reduce the virulence as the survival of infected mice in systemic infection model was increased (Van Dijck et al., 2002). Indeed, the deletion of this gene leads to impaired growth in *C. albicans*, and other species, and the gene product itself is considered as a potential target for antifungal therapy (Van Dijck et al., 2002; Pianalto and Alspaugh, 2016).

The transformant 3I5 carried a gene library plasmid with a complete *ADH1* open reading frame in the sense direction. It proved to be sensitive to fluconazole, amphotericin B, peptides, SDS, and reactive oxygen species. The *ADH1* gene is encoding alcohol dehydrogenase, responsible for the conversion of acetaldehyde to ethanol. In *C. albicans*, the expression of the *ADH1* and azole resistance is inversely correlated in clinical isolates (Siikala et al., 2011). On the contrary, increased abundance of the Adh1 protein was previously observed in an azole resistant strain of *C. glabrata* (Rogers et al., 2006). Since we do not have the data on the *ADH1* expression in 3I5 strain, further study is needed.

Due to their low toxicity, the peptidomimetics are potent antifungal drugs (Larsen et al., 2015). A mode of action of these peptides is likely the interaction with sphingolipids, as determined by the analysis of deletion mutants’ library in *S. cerevisiae* (Larsen et al., 2015). Larsen et al. (2015) pointed out that higher resistance in pathogenic yeasts (*C. albicans* and *C. glabrata*) to these compounds might be due to the secretion of extracellular proteases, which sequester or degrade the antifungals. One of the isolated *C. glabrata* antisense plasmids carried part of the CAGL0J02464g gene, whose orthologs have SUMO (Small Ubiquitin-like Modifier)-specific isopeptidase and protein deSUMOylation activities. The encoded isopeptidase could be important for the resistance to peptidomimetics in this yeast and could be involved in their direct cleavage, in support of the hypothesis proposed by Larsen et al. (2015). In addition, fragments of two putative aminoacyl-tRNA genes were found with our system affecting the peptidomimetics resistance. We hypothesize that they could directly interact with peptidomimetics or their targets by acylating them. Aminoacyl-tRNA dependent acylation is involved in several cellular processes and resistance to antifungal peptides reported in other species (Raina and Ibba, 2014). Acylation of membrane lipids is known to change the membrane surface and subsequently the affinity of the membrane to antifungal peptides in other species (Ernst and Peschel, 2011; Raina and Ibba, 2014). The direct acylation of macromolecules using aminoacyl-tRNA can change their activity, recognition or directs them to degradation (Katz et al., 2016).

Taking a step further, we validated the importance of one of the identified target in the macrophage model. We confirmed the vital importance of the *C. glabrata* CAGL0K11968g gene affecting colony pH and vacuolar function with this screening method. With the antisense construct for this gene the *C. glabrata* strain was less viable upon exposure to human macrophages.

We have thus established a working RNAi system for the investigation of *C. glabrata* pathobiology. Our initial testing showed that the system can be used to interfere with the expression of a broad range of genes, including those assumed to be essential for *C. glabrata*. The RNAi strains can be used in *in vitro* stress tests and in interaction with immune cells, which will be invaluable in attributing functions to the many genes of *C. glabrata*, which are unannotated so far. Our screening of a RNAi library with genome fragments is a first step into that direction. Especially, as this method enables us to tackle new genes, which are central for fungal growth and survival, we believe that it will allow us to find important new potential targets for *C. glabrata* antifungals, a yeast that is notorious for its inherent and acquired resistances.

## Materials and Methods

### Growth Conditions

All strains used in this study were grown in rich medium (YPD [yeast extract 1%, peptone 2%, glucose 2%, Bactoagar 2%] or synthetic minimal medium (YNB [yeast nitrogen base without amino acid and ammonium sulfate] 1.9 g/L, glucose 2%, ammonium sulfate 0.5%) at 25°C, unless stated otherwise. For the uracil-deficient mutant BG14, 50 mg/L uracil was added to the YNB medium. For the lysine-deficient mutants, 40 mg/L lysine was added to the minimal medium. *C. glabrata* transformants carrying plasmids with the *LYS2* gene as a selectable marker were selected and propagated on YNB medium. For *C. glabrata* transformants carrying the *Streptoalloteichus hindustanus ble* gene as a selective marker, 200 μg/ml of zeocin was added to the YPD medium. For the induction of the *CUP1* promoter, 0.05 mM CuSO_4_ was added to the YNB medium.

For gene library yeast transformants different compounds were added to YNB medium as follows: amphotericin B (0.05 and 0.25 μg/ml), fluconazole (50 and 400 mg/L), SDS (0.01 and 0.0025%), hydrogen peroxide (3 and 12 mM), menadione (0.02 and 0.06 mM), and antifungal peptidomimetics (peptide 6 [H-Arg-Tbt-Arg-Phe-NH2], 9 [H-Arg-Tbt-Arg-hPhe-NH2], and 11 [H-Arg-Tbt-Arg-[NPhe]-NH2] (Larsen et al., 2015) at 25 μg/ml. Gene library transformants were propagated at 37°C.

### RNA Extraction and qRT-PCR

Total RNA was extracted from the *C. glabrata* cultures grown in selective media (supporting plasmids propagation) using the PureLink RNA Mini Kit (Thermo Fisher Scientific). The concentration and purity of RNA were determined by NanoDrop spectrophotometer. The isolated RNA was treated with DNase I (RNase-Free DNase Set, Qiagen) according to the manufacturer’s recommendations. The RNA integrity was checked by electrophoresis using precast RNA MOPS agarose gels (Sigma-Aldrich). Five microgram of pure RNA was used for the synthesis of cDNA. The SuperScript III Reverse Transcriptase kit with RNaseOUT Ribonuclease Inhibitor and random primers (Thermo Fisher Scientific) was used. The cDNA produced was used as a template with gene-specific primers in qRT-PCR reactions with the SYBR GreenER qPCR SuperMix (Thermo Fisher Scientific). qRT-PCRs were run in duplicate in the RotorGene 2000 cycler (Corbett Research) under the conditions specified by Thermo Fisher Scientific. The take off and amplification values were obtained using the RotorGene 2000 software. The β-actin gene was treated as the endogenous reference gene (housekeeping gene), while Y1848 was used as untreated strain. Primer pairs for the 3′-region of the gene ORFs of interest of *C. glabrata* were used in qRT-PCR experiments as follows for the: *ACT1* (OP33 and OP34; ACT1-1 and ACT1-2), *ADE2* (OP31 and OP32), *URA3* (OP27 and OP28), *PUP1* (947-1 and 947-2), CAGL0L00157g (157-1 and 157-2), CAGL0E00231g (231-3 and 231-4), CAGL0G05335g (5335-3 and 5335-4), CAGL0E00539g (539-1 and 539-2), CAGL0K11968g (968-1 and 968-2), CAGL0H00891g (891-1 and 891-2), CAGL0A01430g (1430-3 and 1430-4), CAGL0H07623g (7623-1 and 7623-2), and CAGL0I11011g (11-1 and 11-2) (Supplementary Table S1). The gene expression fold change was calculated by the ΔΔCt method (Livak and Schmittgen, 2001).

### Macrophage Culture and Infection With Yeast

Human monocyte-derived macrophages (hMDMs) were prepared according to the protocol used before (Seider et al., 2011, 2014). Monocytes were isolated from human peripheral blood (donated by healthy volunteers with written consent) with CD14 magnetic beads by automated cell sorting (autoMACs, MiltenyiBiotec). CD14-positive monocytes differentiated to M2 macrophages for 7 days in RPMI1640 media with L-glutamine (Thermo Fisher Scientific) with 10% heat-inactivated FCS (Bio&Sell GmbH), and with 50 ng/ml recombinant human macrophage colony stimulating factor (rh M-CSF; Immunotools). Adherent MDMs were detached with 10 mM EDTA in PBS and seeded in 96-well plates (4 × 10^4^ hMDMs/well) in RPMI with 50 ng/ml rh M-CSF and 10% FCS and incubated overnight. Prior macrophage infection yeast cells from a stationary YNB culture were washed three times with PBS. M2 macrophages were infected by adding the yeast cells at multiplicity of infection of one (MOI 1) in RPMI w/o FCS. The cells were further diluted and incubated for 3 h at 5% CO_2_ and 37°C. Unattached yeast cells were removed by washing the macrophages two times with 60 μl PBS. Next, 20 μl of 0.5% Triton X-100 was added to lysate the macrophages, and incubated 10 min under gentle shaking. After the incubation, cells were diluted, plated on YPD, and incubated at 37°C for 1 day. After incubation, the yeast CFU were counted. The reference strain Y1848 carrying empty vector was used to normalize obtained data and was set to 100% survival.

### Robotics Screening

Individual transformants of the *C. glabrata* RNAi strain (Y1662) containing the gene library plasmids were grown overnight in flat bottom polystyrene 96-well plates in 200 μl of liquid YNB at 37°C (12 plates in total). The next day, yeast transformants were plated onto the solid media YNB supplemented with antifungal drugs [amphotericin B (0.05 and 0.25 μg/ml) or fluconazole (50 and 400 μg/ml)], ROS generating compounds [menadione (0.02 and 1 0.06 mM) and hydrogen peroxide (3 and 12 mM)], surfactant (SDS 0.01 and 0.0025%) and a pH indicator (bromocresol green 0.01 g/ml, YNB medium pH was adjusted to 4.5) using a robotic system (Siger RoToR HDA robot). Each 96-well plate liquid culture was plated on solid medium in duplicate, and four were combined onto one 384 format solid medium plate. The colonies growth was scored by the colony size in pixels from the digital images of the plates during 96 h of incubation.

### Vacuolar Staining and Fluorescent Microscopy

Cells were re-suspended in 10 mM HEPES buffer pH 7.4 supplemented with 5% glucose. The fluorescent dye CMAC-Ala-Pro (7-amino-4-chloromethylcoumarin, l-alanyl-l-proline amide, Yeast Vacuolar Marker Sampler Kit [Thermo Fisher Scientific]) was added to the cell suspension at 100 μM and then incubated in the dark for 30 min. The staining was visualized by fluorescent microscopy (automated inverted wide-field microscope Observer Z1 [Carl Zeiss] equipped with a sCMOS camera).

### Statistical Analysis

The software packages R (Version 1.1.463– ©2009–2018 RStudio, Inc.), JMP^®^, Pro 13.0.0 (SAS Institute Inc., Cary, NC, United States, 1989–2019) and Minitab^®^18.1, were used to analyze the obtained data.

## Dedication

In loving memory of JP who sadly deceased on May 18, 2014.

## Data Availability

All datasets generated for this study are included in the manuscript and/or the Supplementary Files.

## Ethics Statement

The protocols for the experiments with macrophages were approved by the ethical commissions of the Lund University and the Hans Knöll Institute, Jena.

## Author Contributions

OI and JP designed the study. OI, KA, KK, KB, and TS developed the RNAi tool. LK, SB, MS, BH, TH, BG, and OI performed the macrophage experiments. CB, KF, JS, BR, and OI analyzed the gene library. OI, KA, WK, and JP wrote the manuscript. All authors commented on and reviewed the manuscript.

## Conflict of Interest Statement

The authors declare that the research was conducted in the absence of any commercial or financial relationships that could be construed as a potential conflict of interest.

## Footnotes

1 www.yeastgenome.org
