# Supplementary material for: RNAi as a Tool to Study Virulence in the Pathogenic Yeast Candida glabrata
Source: Front Microbiol. 2019 Jul 24;10:1679. doi: 10.3389/fmicb.2019.01679 (PMC6667738; doi:10.3389/fmicb.2019.01679)
Supplement: Supplementary file 1 [file Data_Sheet_1.pdf]

Supplementary material

1 Supplementary data

Selection of lys2 mutants of C. glabrata

The *lys2* mutants of BG2 and CBS 138 strains of *C. glabrata* were selected by spontaneous mutagenesis. The corresponding strains were plated on the minimal medium supplemented with YNB, 2% glucose, 40 mg/L of lysine, 1 g/L of α-aminoadipate (as nitrogen source), and 2% agar. Since WT strains are unable to grow on this medium, mutant colonies were obtained by positive selection using the ability of *lys2* mutants to utilize α-aminoadipate as a nitrogen source (Chattoo et al., 1979). The obtained colonies were first analysed for lysine auxotrophy, after which the complementation test was performed by transformation with the plasmid vector carrying *LYS2* gene of *C. glabrata.* The transformation efficiency was 5.6×10^3 transf/μg of DNA. Verified *lys2* mutants, Y1636 (BG2 origin) and Y1637 (CBS 138 origin), were further used as recipient strains to express the *S. castellii DCR1* and *AGO1* genes under *C. glabrata* promoters.

Plasmid construction

Oligonucleotides used in this study are listed in Table S1. For evaluation of different promoter activities in *C. glabrata*, the reporter plasmids P1163-P1169 were constructed on the basis of pGRB2.0 (Table S2). The promoters of interest were fused to the reporter gene of *Drosophila melanogaster* (Dm) *dNK*, encoding deoxyribonucleoside kinase [Munch-Petersen et al., 2000], and the *C. glabrata* terminator *CYC1* using overlap PCR. Fusion products were cloned into the *BamHI* site of the plasmid pGRB2.0. For fusion 1, *C. glabrata* promoter *TEF1* was amplified with primers Len1 and Len2; the Dm dNK ORF - with Len3 and Len4; the *C. glabrata* terminator *CYC1* - with Len5 and Len6. Primers Len1 and Len6 were used for overlap PCR to fuse three fragments together. For fusion 2, the *C. glabrata* promoter *TDH3* was amplified with primers Len7 and Len8; the Dm dNK ORF - with Len9 and Len4; the *C. glabrata* terminator *CYC1* - with Len5 and Len6. Primers Len7 and Len6 were used for overlap PCR to fuse three fragments together. For fusion 3, the *S. cerevisiae* promoter *TEF1* was amplified with primers Len10 and Len11; the Dm dNK ORF - with Len12 and Len4; the *C. glabrata* terminator *CYC1* - with Len5 and Len6. Primers Len10 and Len6 were used for overlap PCR to fuse three fragments together. For fusion 4, the *S. cerevisiae* promoter *GAL1* was amplified with primers Len13 and Len14; the Dm dNK ORF - with Len15 and Len4; the *C. glabrata* terminator *CYC1* - with Len5 and Len6. Primers Len13 and Len6 were used for overlap PCR to fuse three fragments together. For fusion 5, the *C. glabrata* promoter *CUP1* was amplified with primers Len16 and Len17; the Dm dNK ORF - with Len18 and Len4; the *C. glabrata* terminator *CYC1* - with Len5 and Len6. Primers Len16 and Len6 were used for overlap PCR to fuse three fragments together. For fusion 6, the *C. glabrata* promoter *PGK1* was amplified with primers Len19 and Len20; the Dm dNK ORF - with Len21 and Len4; the *C. glabrata* terminator *CYC1* - with Len5 and Len6. Primers Len19 and Len6 were used for overlap PCR to fuse three fragments together. For fusion 7, the *C. glabrata* promoter *ADH1* was amplified with primers Len22 and Len23; the Dm dNK ORF - with Len24 and Len4; the *C. glabrata* terminator *CYC1* - with Len5 and Len6. Primers Len22 and Len6 were used for overlap PCR to fuse three fragments

together.

P1028 was constructed based on pUC19. The *C. glabrata LYS2* gene was amplified with primers Len28 and Len29 and cloned into *Bam*HI site of pUC19.

P1062 was constructed based on pUC19. The *S. hindustanus ble* gene was amplified from pPICZB (Invitrogen) using primers Len44 and Len45 and ligated to *Eco*RI/*Bam*HI-treated pUC19. The promoter *TEF1* of *C. glabrata* was amplified with primers Len71 and Len32. *S. castellii DCR1* ORF was amplified with primers Len33 and Len34. The *C. glabrata* terminator *CYC1* was amplified using primers Len35 and Len72. Primers Len71 and Len72 were used in overlap PCR reaction to fuse together promoter with ORF and terminator that was subsequently digested with *Bam*HI and cloned into the corresponding site of pUC19. The *C. glabrata* promoter *TDH3* was amplified with primers Len68 and Len39. *S. castellii AGO1* ORF was amplified with primers Len40 and Len41. The *C. glabrata* terminator *TDH3* was amplified using primers Len42 and Len69. Primers Len68 and Len69 were used in overlap PCR reaction to fuse together promoter with ORF and terminator that was further digested with *Hind*III and cloned into corresponding site of pUC19 carrying *DCR1* expression cassette resulting in the plasmid P1062 (Table S2).

P1030 was constructed based on P1028. The *C. glabrata* promoter *CUP1* was amplified with primers Len48 and Len49. The *URA3* hairpin part was constructed as it was described by Drinnenberg *et al.,* 2009, where the sense region is of 339 bp size was followed by a 79 bp region representing a hairpin loop and subsequent 339 bp antisense regions. Therefore, the middle part of the gene of 418 bp (sense region + 79 bp of the downstream region [hairpin loop]) was amplified with primers Len50 and Len51; the antisense region of 339 bp (reverse complement of sense region) was amplified with primers Len52 and Len53. The *C. glabrata* terminator *CYC1* was amplified with primers Len54 and Len55. In overlap PCR primers Len 48 and Len51 were used to fuse promoter and sense region with the loop; primers Len52 and Len55 were used to fuse antisense region and terminator. Subsequently, the two fragments obtained by overlap PCR were treated with *SacI* and *SalI* and cloned into *SacI* site of P1028.

P1029 was constructed based on P1028. The *C. glabrata* promoter *CUP1* was amplified with primers Len48 and Len56. The antisense region of the *C. glabrata URA3* gene of 339 bp size (middle part of the *URA3*), that was used for *URA3* hairpin construction in P1030 was amplified with primers Len57 and Len53. The *C. glabrata* terminator *CYC1* was amplified with primers Len54 and Len55. Primers Len48 and Len55 were used for overlap PCR to fuse the three fragments together.

P1066 was constructed based on P1028. The *C. glabrata* promoter *CUP1* was amplified with primers Len58 and Len59. The *ADE2* hairpin construct was constructed similarly to the *URA3* hairpin. 418 bp (sense region with 79 bp of the downstream region [hairpin loop]) of the 3'-part of the gene was amplified with primers Len60 and Len61; the antisense region of 339 bp (reverse complement of sense region) was amplified with primers Len62 and Len63. The *C. glabrata* terminator *CYC1* was amplified with primers Len64 and Len65. In overlap PCR primers Len58 and Len61 were used to fuse promoter and sense region with the loop; primers Len62 and Len65 were used to fuse antisense region and the terminator. Subsequently, the two fragments obtained by overlap PCR were treated with *Sac*I and *SalI* and cloned into *SacI* site of P1028.

P1065 was constructed based on P1028. The *C. glabrata* promoter *CUP1* was

amplified with primers Len58 and Len66. The antisense region of the *C. glabrata ADE2* gene of 339 bp size that was used for *ADE2* hairpin construction in P1066 was amplified with primers Len67 and Len63. The *C. glabrata* terminator *CYC1* was amplified with primers Len64 and Len65. Primers Len58 and Len65 were used for overlap PCR to fuse the three fragments together.

P1061 was constructed based on P1028. The DNA fragment carrying the 18s rDNA of *C. glabrata* of 1.8 kb was amplified with KM1 and KM4, treated with both *Sal*I and *Sph*I and cloned into *Sal*I- and *Sph*I-digested P1028.

P1125 was constructed based on P1061. The *C. glabrata* promoter *PGK1* was amplified with KL1 and KL57, the antisense fragment of the CAGLM12947g gene of 345 bp was amplified with KL58 and KL55, the *C. glabrata* terminator *CYC1* was amplified with KL56 and KL8. Three fragments were fused in overlap PCR using the KL1 and KL8 primers pair and cloned into P1125 digested with *Sph*I.

P1151 was constructed based on P1061. The *C. glabrata* promoter *PGK1* was amplified with KL1 and KL51, the sense fragment of the CAGLM12947g gene of 421 bp (including hairpin loop) was amplified with KL52 and KL53, the antisense fragment of the CAGLM12947g gene of 345 bp was amplified with KL54 and KL55, the *C. glabrata* terminator *CYC1* was amplified with KL56 and KL8. The promoter and sense fragment were fused in overlap PCR using primers KL1 and KL53. The antisense fragment and terminator fragments were fused in overlap PCR using primers KL54 and KL8. The obtained overlap fragments were treated with *Sph*I and *Bgl*II and cloned into *Sph*I site of P1061.

P1225 was constructed based on P1028. P1028 was digested with *Pst*I and ligated with *Pst*I fragment of 2.5 kb carrying ARS-like sequence (the *S. cerevisiae COXII* gene) from pMIR4 (Hanic-Joyce and Joyce, 1998) resulting in plasmid P1225.

P1226 was constructed based on P1225. For this purpose, *C. glabrata* promoter *TEF1* was amplified with primers prTEF1_F and prTEF1_R, the *C. glabrata* terminator *CYC1* was amplified with primers terCYC1_F and terCYC1_R. The amplified promoter and terminator were further fused in overlap PCR using the primer pair prTEF1_F and terCYC1_R. The obtained fusion fragment was digested with *Bgl*II and cloned into P1225.

Deoxyribonucleoside kinase (dNK) enzyme assay

Cell samples grown in the minimal medium YNB with 2% glucose up to OD_{600} of 2.0 were harvested using low-speed centrifugation (1699 *g*) and washed at 4 °C with PBS buffer pH 7.4 containing 2.5 mM DTT, 10% glycerol, 0.1% Triton X-100 and cOmplete™ Protease Inhibitor Cocktail (Roche).

Cells were diluted in the corresponding buffer 2-fold and cell-free extracts were prepared using glass beads with vigorous vortexing at 4 °C. Unbroken cells and cell debris were removed using centrifugation at 20817 *g*. The supernatant was used as a cell-free extract for the enzyme assay. The Dm dNK activity was determined by initial velocity measurements based on triplicates by the DE-81 filter paper assay using ^{3}H-dThd as substrate and liquid scintillation (as described by Munch-Petersen *et al*., 2000). The standard assay conditions used ATP and ^{3}H-labeled acceptor substrate. One unit (U) of Dm dNK activity is defined as 1 µmol of the ^{3}H-dTMP product formed per min. The protein concentration was measured by Bradford method (Bradford, 1976).

Selection of promoters for gene expression in C. glabrata

C. glabrata promoters *TEF1*, *TDH3*, *CUP1*, *PGK1*, *ADH2* and *S. cerevisiae* promoters *TEF1*, *GAL1* were cloned and fused to the *dnk* gene of *D. melanogaster* coding for deoxyribonucleoside kinase that was used as a reporter gene in this study. Resulting replicative plasmids (Table S2) were used to transform the *C. glabrata ura3* mutant BG14 (Y1638, Table 1), and the activity of deoxyribonucleoside kinase expressed from the promoters of interest was tested (Fig. S1). Judging from the activity of the reporter gene expressed from different promoters, the constitutive promoters *TDH3*, *TEF1* and *PGK1* of *C. glabrata* displayed the highest activity in glucose medium, and the activity of the *C. glabrata* promoter *TEF1* was 6.6-times higher compared to the promoter *TEF1* of *S. cerevisiae*. The promoter *CUP1* was proven to be regulated by copper ions and had the highest activity under induction conditions (Fig. S1). For this reason, *C. glabrata* promoters *TDH3* and *TEF1* were selected for the expression of the *S. castellii DCR1* and *AGO1* genes and the *C. glabrata* promoter *CUP1* and *PGK1* were selected for the expression of silencing constructs.

Yeast transformants stability test

Stable transformants were selected after alternating cultivation in nonselective and selective media (in case of the *LYS2* gene as a selective marker: YPD and YNB were used; in case of the *Sh ble* marker YPD and YPD with zeocin were used). In this procedure, cells from single colonies of yeast transformants were inoculated into nonselective medium and grown for approximately 60 generations at 25 °C. Later, the cultures were cloned to obtain single colonies on nonselective medium with subsequent replica-plating on the selective medium. The transformants that remained prototrophic or zeocin resistant after this type of cultivation were selected and checked by PCR for the presence of the introduced DNA constructs.

2 Supplementary references

1. Munch-Petersen, B., Knecht, W., Lenz, C., Søndergaard, L., and Piskur, J. (2000). Functional expression of a multisubstrate deoxyribonucleoside kinase from *Drosophila melanogaster* and its C-terminal deletion mutants. J. Biol. Chem. 275, 6673-6679. doi: 10.1074/jbc.275.9.6673
2. Bradford, M. M. (1976). A rapid and sensitive method for the quantitation of microgram quantities of protein utilizing the principle of protein-dye binding. Anal. Biochem. 72, 248-254. doi: 10.1016/0003-2697(76)90527-3
3. Chattoo, B.B., Sherman, F., Azubalis, D.A., Fjellstedt, T.A., Mehnert, D., and Ogur, M. (1979). Selection of *lys2* Mutants of the Yeast *SACCHAROMYCES CEREVISIAE* by the Utilization of alpha-AMINOADIPATE. Genetics. 93(1), 51-65.
4. Drinnenberg, I.A., Weinberg, D.E., Xie, K.T., Mower, J.P., Wolfe, K.H., Fink, G.R., and Bartel, D.P. (2009). RNAi in budding yeast. Science. 326(5952), 544-550. doi: 10.1126/science.1176945

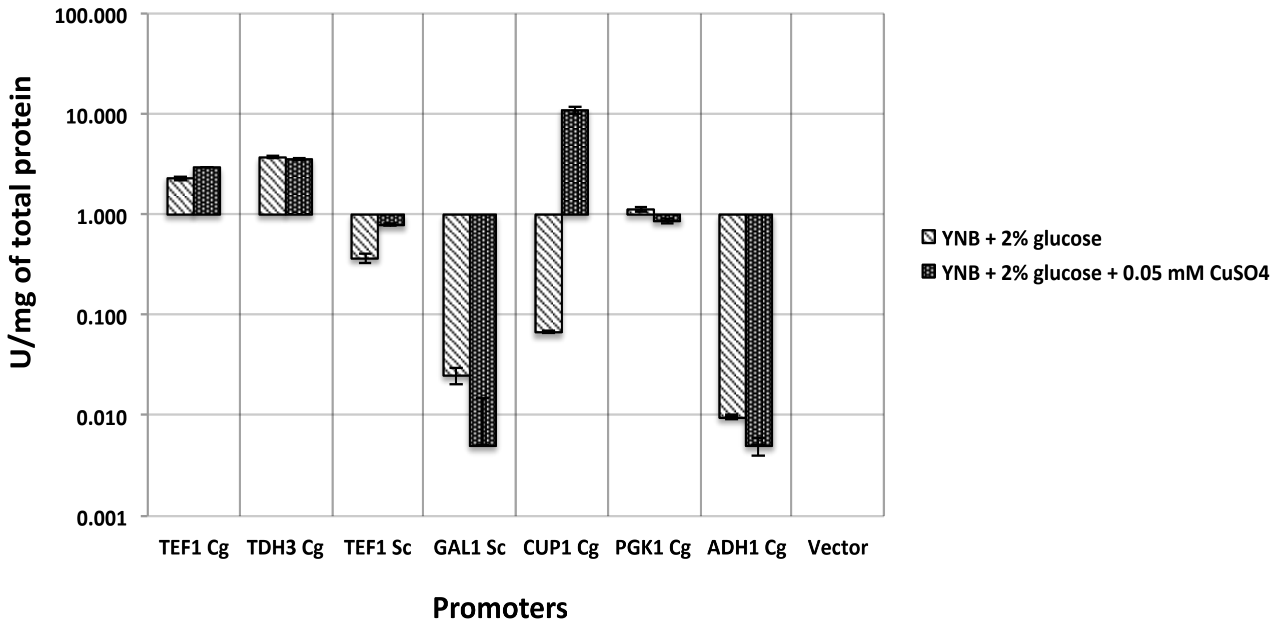

Figure S1. ***D. melanogaster*** **dNK activity expressed form different promoters in** ***C. glabrata.*** Yeast transformants, carrying the reporter gene expressed from *C. glabrata* (Cg) or *S. cerevisiae* (Sc) promoters, were grown in minimal medium YNB with 2% glucose with or without copper ion supplementation prior to the dNK enzyme assay. The *error* bars represent standard deviation of two replicates. The Y-axis has a logarithmic scale.

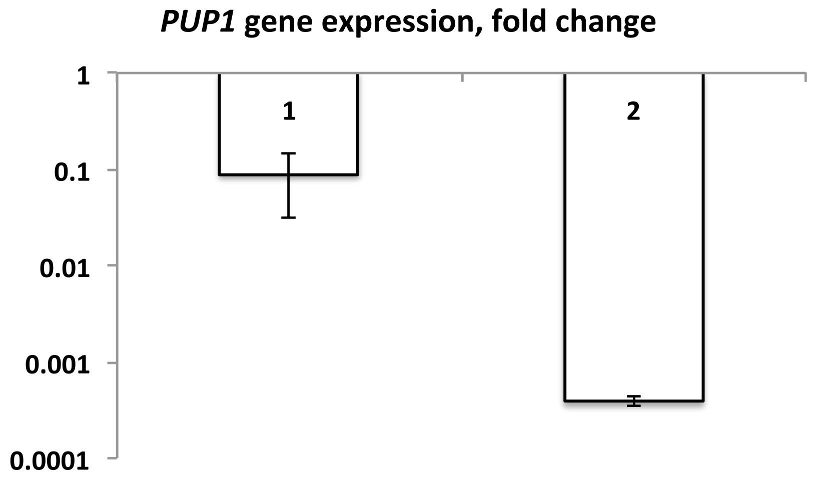

Figure S2.
Expression analysis of *PUP1* gene expression in RNAi transformants performed by qRT-PCR. Strains: 1 – *PUP1* antisense, 2 – *PUP1* hairpin. Corresponding primer pairs were used with cDNA: *ACT1* gene (ACT1-1 and ACT1-2), *PUP1* (947-1 and 947-2). Strains carrying empty vector (Y1848) was used as untreated control. Y-axis has logarithmic scale.

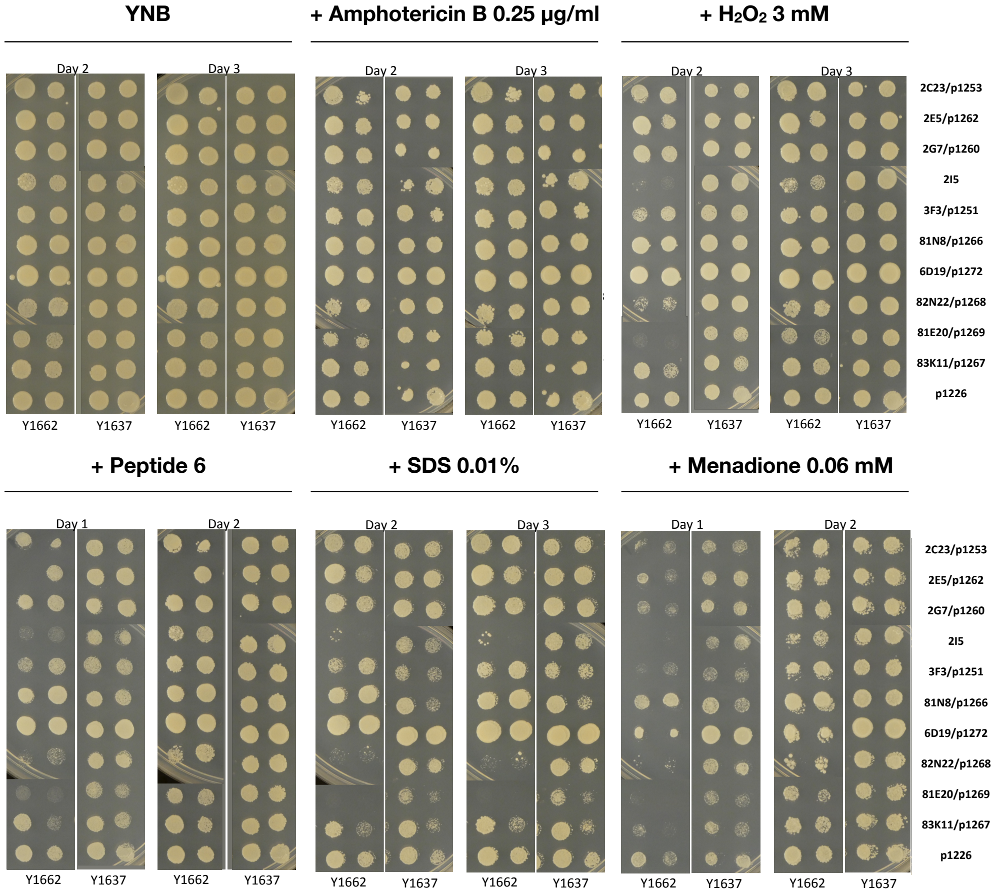

Figure S3.
Phenotypic profiling of antisense plasmids transformants of RNAi and wild type strain. RNAi strain was Y1662, and the wild type was Y1637, which were newly re-transformed with plasmids. Antisense plasmids: 1 – 2C23 (CAGL0L00157g gene), 2 – 2E5 (CAGL0E00231g gene), 3 – 2G7 (CAGL0I11011g gene), 4 – 2I5 (CAGL0G05335g gene), 5 – 3F3 (CAGL0K11968g gene), 6 – 81N8 (CAGL0A01430g gene), 7 – 6D19 (CAGL0E00539g gene), 8 – 82N22 (CAGL0H00891g gene), 9 – 81E20 (CAGL0G05335g gene), 10 – 83K11 (CAGL0H07623g gene), 11 – empty vector (P1226).

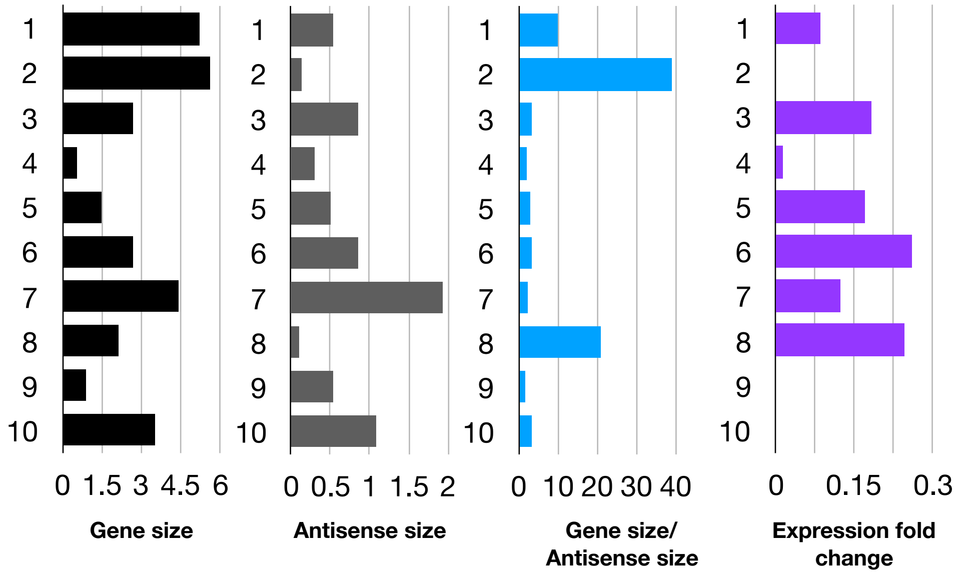

Figure S4.
The target-gene size, antisense-construct size, their ratio, and expression fold-change.
1 – 2C23 (CAGL0L00157g gene), 2 – 2E5 (CAGL0E00231g gene), 3 – 81E20 (CAGL0G05335g gene), 4 – 6D19 (CAGL0E00539g gene), 5 – 3F3 (CAGL0K11968g gene), 6 – 2I5 (CAGL0G05335g gene), 7 – 82N22 (CAGL0H00891g gene), 8 – 81N8 (CAGL0A01430g gene), 9 – 83K11 (CAGL0H07623g gene), and 10 – 2G7 (CAGL0I11011g gene).

Table S1.

Oligonucleotides used in this study.

**Name**	Sequence 5'-3'
Len1	TATGGATCCCGCTGATCTCTGTGCGTGTCCC
Len2	CAGGATGCTGCCTCCGCCATTGTTGATTATTGTATGTG
Len3	CACATACAATAATCAACAATGGCGGAGGCAGCATCCTG
Len4	CCTTTTGTTTTCATAGGAGCTTATCTGGCGACCCTCTGGC
Len5	GCCAGAGGGTCGCCAGATAAGCTCCTATGAAAACAAAAGG
Len6	CGGGGGATCCCTTAGGAGTTTTGTAGTATATGTACATGTAG
Len7	CACGGATCCACTGTGCCACCAATAAATGTATGGGTG
Len8	GGATGCTGCCTCCGCCATTTTTGATGTTATGTTTGTTGTG
Len9	CACAACAAACATAACATCAAAAATGGCGGAGGCAGCATCC
Len10	GCCGCGGGATCCTGCTAAACTAAATAGGCATTTG
Len11	GGATGCTGCCTCCGCCATTTTGTAATTAAAACTTAGATTAG
Len12	CTAATCTAAGTTTTAATTACAAAATGGCGGAGGCAGCATCC
Len13	CCCGGATCCCCAGCAAAGTGAATTACCGAATC
Len14	GGATGCTGCCTCCGCCATTATAGTTTTTTCTCCTTGAC
Len15	GTCAAGGAGAAAAAACTATAATGGCGGAGGCAGCATCC
Len16	TCTGGATCCCGGAGCACCTGGCTGATTTACC
Len17	GGATGCTGCCTCCGCCATTGTGTTTGTTTTTGTATGTGTTTG
Len18	CAAACACATACAAAAACAAACACAATGGCGGAGGCAGCATCC
Len19	CCGCGGGATCCCACGATCATGTTTGAAATTGTAG
Len20	GGATGCTGCCTCCGCCATTATCGAATAGATGTATGTATG
Len21	CATACATACATCTATTCGATAATGGCGGAGGCAGCATCC
Len22	CCCGGATCCCATACTGTTACACGCACAAGCATG
Len23	GGATGCTGCCTCCGCCATTGTTTATGTGTTTTTTGCAG
Len24	CTGCAAAAAACACATAAACAATGGCGGAGGCAGCATCC
Len28	TCCGGATCCGTGGGAACCCAAGTACATGGATG
Len29	CCCGGATCCATGGCAAAGTGGAGATAGAGTGC
Len32	GATCGGCGCTTTTTTCTCTATTCATTGTTGATTATTGTATGTG
Len33	CACATACAATAATCAACAATGAATAGAGAAAAAAGCGCCGATC
Len34	CCTTTTGTTTTCATAGGAGCTCACAGATTGTTGCAATGC
Len35	GCATTGCAACAATCTGTGAGCTCCTATGAAAACAAAAGG
Len39	CTCCGAATTGGATGACATTTTTGATGTTATGTTTGTTGTG
Len40	CACAACAAACATAACATCAAAAATGTCATCCAATTCGGAG
Len41	GTTATAATAAATATCTTTTAAGATTTTCATATGTAGTACATGATG
Len42	CATCATGTACTACATATGAAAATCTTAAAAGATATTTATTATAAC
Len44	CCCGAATTCCACCATAGCTTCAAAATGTTTC
Len45	CTCGGATCCTTCTCAAGCAAGGTTTTCAG
Len48	TATGAGCTCCGGAGCACCTGGCTGATTTAC
Len49	CTTGGCTGCCATTTCTTTCACATTGTGTTTGTTTTTGTATG

Len50	CATACAAAAACAAACACAATGTGAAAGAAATGGCAGCCAAG
Len51	ATAGTCGACTGTTGACCAAGTGCATCGCCTTTATC
Len52	CACGTCGACCACCCATATCCTTTTGAGCAATG
Len53	CTTTTGTTTTCATAGGAGCTTATGAAAGAAATGGCAGCC
Len54	GGCTGCCATTTCTTTCATAAGCTCCTATGAAAACAAAAG
Len55	CCCGAGCTCCTTAGGAGTTTTGTAGTATATG
Len56	CTCAAAAGGATATGGGTGCATTGTGTTTGTTTTTGTATG
Len57	CATACAAAAACAAACACAATGCACCCATATCCTTTTGAG
Len58	TTTGAGCTCCGGAGCACCTGGCTGATTTAC
Len59	GTTTTTGGACTTCCTTCCATTGTGTTTGTTTTTGTATGTG
Len60	CACATACAAAAACAAACACAATGGAAGGAAGTCCAAAAAC
Len61	TATGTCGACCACAAGTGGCACACATTGTTTTTGTG
Len62	CGCGTCGACTCATTACAGATAATTCCTTCTTAAAG
Len63	CTTTTGTTTTCATAGGAGCCTAGAAGGAAGTCCAAAAAC
Len64	GTTTTTGGACTTCCTTCTAGGCTCCTATGAAAACAAAAG
Len65	CCGGAGCTCCTTAGGAGTTTTGTAGTATATG
Len66	GAAGGAATTATCTGTAATGACATTGTGTTTGTTTTTGTATGTG
Len67	CACATACAAAAACAAACACAATGTCATTACAGATAATTCCTTC
Len68	CCCAAGCTTCTGTGCCACCAATAAATGTATG
Len69	GCCGCAAGCTTGATTCTTCTTATAAAATGATATTTTAAATAC
Len71	TTTGGATCCCGCTGATCTCTGTGCGTGTCC
Len72	CCGGGATCCCTTAGGAGTTTTGTAGTATATGTACATG
OP27	AGGTATCGTCACTGGTTTAAAGCAAGGTGC
OP28	TCTAGACCGACACCAGGAGTCATTATTAGCC
OP31	AGACAAGGAACTTCAAATATGTGAGAGGGCC
OP32	ATAACACCAACTAAAGGTTTCTTACTTCCTCCGC
OP33	GAATTGCCAGATGGTCAAGTCATCACCATCG
OP34	GGTAATTTCCTTTTGCATTCTTTCAGCAATACCTG
KM1	TATGTCGACTATCTGGTTGATCCTGCCAGTAG
KM4	TTTGCATGCTAATGATCCTTCCGCAGGTTCAC
KL1	CACGCATGCCACGATCATGTTTGAAATTGTAG
KL8	CCCGCATGCCTTAGGAGTTTTGTAGTATATG
KL51	GTACCTGTATGCGTAGGCATTATCGAATAGATGTATG
KL52	CATACATCTATTCGATAATGCCTACGCATACAGGTAC
KL53	GGGAGATCTATTTGGTGCCAAAATTTATCAGCC
KL54	CTCAGATCTGGTCTGGGTTTGGAAACTTTCTG
KL55	CTTTTGTTTTCATAGGAGCTCACCTACGCATACAGGTAC
KL56	GTACCTGTATGCGTAGGTGAGCTCCTATGAAAACAAAAG
KL57	GTTTCCAAACCCAGACCCATTATCGAATAGATGTATG
KL58	CATACATCTATTCGATAATGGGTCTGGGTTTGGAAAC
947-1	CCTAGGACAATCTTACTGGGTGTAGTC
947-2	ATACATAGCCCACCTTGGTGCCGTC
ACT1-1	AACGTTCCAGCCTTCTACGTTTCCATTC
ACT1-2	TTCTCTTTCAGCAGTGGTGGAGAAAGAG

157-1	AGGACAGGTACAACTACTATCTCCACTG
157-2	TGTGTGACCGTTAGAAACCACGGTG
231-3	CCTACCACGATCACCACCACCATC
231-4	CATGAGAAACAACAGCTGTAATCGTATGACC
11-1	AGCACAGTTTCCAACGGCAAGCCTAC
11-2	GTCAGAGCCGGTGATCACGGTG
5335-3	TTGGAACAACAGGTCAACGAATTGGTCAAC
5335-4	TGGGTTACCATAACTCAAGGCATTTGCTG
968-1	GAAGCATTTGAAGGAACTCCAGAAATGATG
968-2	GCTTGTCCAACAGCTTATGCTTCGTAG
1430-3	GAACACTGTGGTGGTGCCCAAATC
1430-4	ATTCAGAGCTTGACGACGAACATCTTCAG
539-1	GATGAGAGATCCCAGAAGGATAAGGAG
539-2	AGTACGCTGTTTCAACAACGGCACG
891-1	GATTCCGAGAATATGAAGAAGACCAAAGTATTG
891-2	TCTCATCGAGAATTCTTTCTCTTTAGCTGC
7623-1	AGCCAAGAGATTCTATTATCGATAGTACCG
7623-2	TAGAGTTGCTTTACCTTCGGTTAGTTCAAC

Table S2.

Plasmids used in this study.

**Plasmid, laboratory designation**	**Description**	**Source**
P1004	pGRB2.0 (Cormack B.), pRS406 backbone, *C .glabrata CEN/ARS*, *URA3*	kindly provided by Cormack B
P1163	pGRB2.0 backbone, carrying expression cassette pr*TEF1* Cg-ORF Dm dNK-ter*CYC1* Cg	This study
P1164	pGRB2.0 backbone, carrying expression cassette pr*TDH3* Cg-ORF Dm dNK-ter*CYC1* Cg	This study
P1165	pGRB2.0 backbone, carrying expression cassette pr*TEF1* Sc-ORF Dm dNK-ter*CYC1* Cg	This study
P1166	pGRB2.0 backbone, carrying expression cassette pr*GAL1* Sc-ORF Dm dNK-ter*CYC1* Cg	This study
P1167	pGRB2.0 backbone, carrying expression cassette pr*CUP1* Cg-ORF Dm dNK-ter*CYC1* Cg	This study
P1168	pGRB2.0 backbone, carrying expression cassette pr*PGK1* Cg-ORF Dm dNK-ter*CYC1* Cg	This study
P1169	pGRB2.0 backbone, carrying expression cassette pr*ADH2* Cg-ORF Dm dNK-ter*CYC1* Cg	This study
P1062	pUC19 backbone, carrying *Sh ble* as selective marker, and expression cassettes: pr*TEF1* Cg-ORF DCR1 Sc-ter*CYC1*, pr*TDH3* Cg-ORF_*AGO1* Sc-ter*TDH3* Cg	This study
P1028	pUC19 backbone, *C. glabrata LYS2* gene	This study

P1029	P1028 backbone, carrying expression cassette pr*CUP1* Cg-*URA3* antisense Cg-ter*CYC1* Cg	This study
P1030	P1028 backbone, carrying expression cassette pr*CUP1* Cg-*URA3* hairpin Cg-ter*CYC1* Cg	This study
P1065	P1028 backbone, carrying expression cassette pr*CUP1* Cg-*ADE2* antisense Cg-ter*CYC1* Cg	This study
P1066	P1028 backbone, carrying expression cassette pr*CUP1* Cg-*ADE2* hairpin Cg-ter*CYC1* Cg	This study
P1061	pUC19 + Cg*LYS2* + Cg18s rDNA	This study
P1125	P1061 backbone, carrying expression cassette Cg pr*PGK1* *CAGL0M12947g* antisense ter*CYC1* Cg	This study
P1151	P1061 backbone, carrying expression cassette Cg pr*PGK1* *CAGL0M12947g* hairpin ter*CYC1* Cg	This study
P1225	P1028 backbone carrying *COXII* gene of *S. cerevisiae* (*ARS*-like sequence)	This study
P1226	P1225 back bone carrying expression cassette with promoter Cg *TEF1* and ter*CYC1* Cg	This study
